# Testosterone Reduces Knee Passive Range of Motion and Expression of Relaxin Receptor Isoforms via 5α-Dihydrotestosterone and Androgen Receptor Binding

**DOI:** 10.3390/ijms15034619

**Published:** 2014-03-17

**Authors:** Firouzeh Dehghan, Sekaran Muniandy, Ashril Yusof, Naguib Salleh

**Affiliations:** 1 Department of Physiology, Faculty of Medicine, University of Malaya, Kuala Lumpur 50603, Malaysia; E-Mail: fir_dhn@yahoo.com; 2 Department of Molecular Medicine, Faculty of Medicine, University of Malaya, Kuala Lumpur 50603, Malaysia; E-Mail: sekaran@um.edu.my; 3 Department of Physiology, Sports Center, University of Malaya, Kuala Lumpur 50603, Malaysia; E-Mail: ashril@um.edu.my

**Keywords:** testosterone, knee ROM, relaxin, Rxfp1 and Rxfp2

## Abstract

Ovarian steroids such as estrogen and progesterone have been reported to influence knee laxity. The effect of testosterone, however, remains unknown. This study investigated the effect of testosterone on the knee range of motion (ROM) and the molecular mechanisms that might involve changes in the expression of relaxin receptor isoforms, Rxfp1 and Rxfp2 in the patella tendon and lateral collateral ligament of the female rat knee. Ovariectomized adult female Wistar rats received three days treatment with peanut oil (control), testosterone (125 and 250 μg/kg) and testosterone (125 and 250 μg/kg) plus flutamide, an androgen receptor blocker or finasteride, a 5α-reductase inhibitor. Duplicate groups received similar treatment however in the presence of relaxin (25 ng/kg). A day after the last drug injection, knee passive ROM was measured by using a digital miniature goniometer. Both tendon and ligament were harvested and then analysed for protein and mRNA expression for Rxfp1 and Rxfp2 respectively. Knee passive ROM, Rxfp1 and Rxfp2 expression were significantly reduced following treatment with testosterone. Flutamide or finasteride administration antagonized the testosterone effect. Concomitant administration of testosterone and relaxin did not result in a significant change in knee ROM as compared to testosterone only treatment; however this was significantly increased following flutamide or finasteride addition. Testosterone effect on knee passive ROM is likely mediated via dihydro-testosterone (DHT), and involves downregulation of Rxfp1 and Rxfp2 expression, which may provide the mechanism underlying testosterone-induced decrease in female knee laxity.

## 1. Introduction

Range of motion (ROM), defined as the movement potential of a joint from full flexion to full extension is controlled by the connective tissue, muscles, tendons, and ligaments [1]. Knee joints connect femur to tibia and fibula through the collateral and anterior cruciate ligaments [2]. Medial and lateral collateral ligaments (MCL and LCL) prevent excessive varus-valgus-forces while anterior cruciate ligament (ACL) prevents knee hyperextension [2]. The patellar tendon, which connects patella to tibia tuberosity stabilizes the patella and also prevents knee hyperextension [3]. Evidence suggests that gender could influence knee laxity. Females have been reported to have greater knee laxity than males [4,5]. Additionally, females have also been reported to have greater ankle laxity [6]. These gender specific differences suggest that male and female sex hormones participate in controlling the generalized joint laxity. So far, studies investigating the relationship between sex hormones and joint laxity mainly focused on the knee and the findings remain inconclusive.

Relaxin, a member of the insulin-like superfamily, has been implicated in the modulation of joint laxity [7]. In primates, relaxin-1 (Rln1), 2 (Rln2), and 3 (Rln3) are the main isoforms [8] while in rats, mice and pigs, Rln1 and Rln3 are the main subtypes [9]. Relaxin increases the joint laxity via upregulating expression and stimulating activity of matrix metalloproteinases (MMPs), collagenases which stimulates collagen degradation and inhibit collagen synthesis by fibroblasts [10]. Relaxin effect is mediated via binding to the relaxin receptor, which consists of the two main isoforms, Rxfp1 and Rxfp2 [11]. Relaxins binding to Rxfp1 and Rxfp2 are species specific. In humans, Rln1 and Rln2 have similar binding affinity to Rxfp1 and Rxfp2, while in rats Rln1 binds weakly to Rxfp2 [12]. Meanwhile, Rln3 has been reported to bind to Rxfp1, Rxfp3, and Rxfp4 [13] while insulin-like factor 3 (Insl3), which is structurally related to relaxin, selectively binds to Rxfp2 [14]. Serum relaxin level fluctuates throughout the reproductive cycle and is markedly increased during pregnancy [15]. Studies have shown a higher plasma relaxin level in pregnant women with pelvic joint instability and increased hip joint laxity [16,17]. While reports have indicated a positive correlation between the serum relaxin concentration and joint laxity [18], relaxin effect on the joint is more reflective in rodents than in humans [9].

Testosterone, an anabolic steroid produced by the testes, ovaries and adrenal glands has serum concentration seven to eight times greater in male than in female [19]. Testosterone promotes the development of secondary male sexual characteristics such as increased in muscle and bone mass and induced male sexual behavior while its active metabolite, 5α-DHT promotes male pattern hair distribution. Additionally, testosterone also stimulates erythropoiesis and increase muscle strength [20]. Despite being produced in low amounts, testosterone is essential for several key reproductive processes in female such as decidualization [21]. The classical effect of testosterone is mediated via a genomic pathway involving the androgen receptor (AR) while a non-genomic pathway mediates its rapid effect [22]. An understanding of the testosterone effect on joint laxity is currently not fully understood. Few studies have suggested that testosterone could influence knee laxity however its exact role remains elusive. Shultz *et al*. [23] reported that testosterone has a positive rather than a negative relationship with changes in knee laxity in the presence of estrogen and progesterone. On the other hand, Rozzi *et al*. [24] reported that male athletes have greater knee joint laxity that female athletes, suggesting a negative influence of testosterone on laxity.

The notion that testosterone decreases joint laxity is further supported by the reported increase in the collagen content of the prostate, breast and capsular tissue and an increase in knee ligament repair strength by testosterone [25]. In view of the fact that relaxin increases while testosterone may decrease joint laxity [26], we hypothesized that testosterone downregulates the expression of the relaxin receptor in the joint, rendering this tissue to be insensitive towards relaxin action. This study therefore aimed to investigate the effect of testosterone on knee joint laxity and changes in relaxin receptor expression under testosterone influence. Additionally, the involvement of the androgen receptor and testosterone active metabolite, 5α-DHT, in mediating testosterone effect was also investigated.

## 2. Results

### 2.1. Passive Knee ROM in Testosterone-Treated Ovariectomised Rats

**Figure 1.**
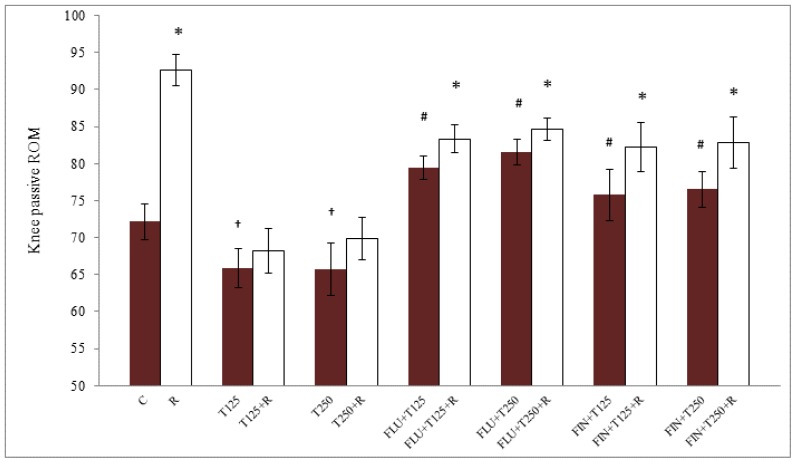
Passive ROM of the rat knee following treatment with peanut oil (control), testosterone, testosterone plus FLU or FIN with and without relaxin. Our findings indicated that treatment with 125 and 250 μg/kg testosterone significantly reduced the knee ROM as compared to control. In the presence of relaxin, knee ROM in the control group was significantly higher than in the testosterone-treated group. Relaxin administration to the group treated with testosterone did not result in a significant increase in the passive knee ROM as compared to without relaxin. The presence of FLU and FIN significantly increase the knee ROM which was further increased following relaxin administration (*p <* 0.05). * *p <* 0.05 as compared to in the absence of relaxin; ^†^ *p <* 0.05 as compared to control; ^#^ *p <* 0.05 as compared to in the absence of FLU or FIN. C: control; R: relaxin; T125: 125 μg/kg/day testosterone; T250: 250 μg/kg/day testosterone; FLU: flutamide; FIN: finasteride.

The degree of knee joint angle (differences between passive flexion and extension) is shown in Figure 1. There was a significant difference in the angle between testosterone-treated and control groups where testosterone caused a decrease in the passive ROM. Testosterone effect was antagonized by flutamide (FLU) and finasteride (FIN). Following treatment with 125 and 250 μg/kg testosterone, the ROM was 6.22° and 6.45° lower respectively than the non-treated group (*p <* 0.05). The degree of knee joint angle in the groups receiving 125 and 250 μg/kg testosterone was greater in the presence of FLU, which were 13.49° and 15.87° higher respectively (*p <* 0.05) as compared to without FLU treatment. The angles in the groups receiving 125 and 250 μg/kg testosterone were also noted to be greater in the presence of FIN, which was 12.84° and 14.82° higher respectively (*p <* 0.05) as compared to without FIN treatment. Knee joint angle was significantly higher in the presence of relaxin in all groups except in the group receiving both doses of testosterone where no difference in the angles was noted. The angle was the highest in the control group, reduced in the presence of testosterone and significantly increased in the presence of FLU and FIN.

### 2.2. Rxfp1 and Rxfp2 mRNA Expression in Patellar Tendon

**Figure 2.**
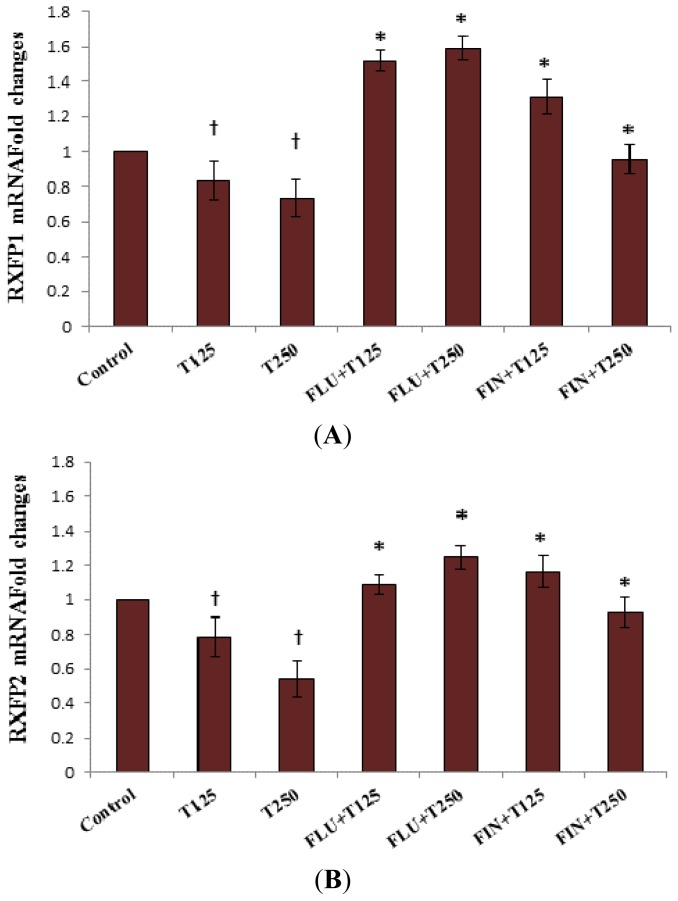
Changes in *Rxfp1* (**A**) and *Rxfp2* (**B**) mRNA expression in the presence of FLU and FIN in the patellar tendon of steroid-replaced ovariectomised rats. Treatment with 125 and 250 μg/kg/day testosterone caused a significant decrease in *Rxfp1* and *Rxfp2* mRNA levels as compared to control. Administration of flutamide and finasteride significantly antagonized the inhibitory effect of testosterone on *Rxfp1* and *Rxfp2* expression with FLU effect relatively higher than FIN for *Rxfp1*, however no significant differences were noted for *Rxfp2* mRNA. ^†^ *p <* 0.05 as compared to control; * *p <* 0.05 as compared to testosterone only treatment for the respective group. T125: 125 μg/kg/day testosterone; T250: 250 μg/kg/day testosterone; FLU: 10 mg/kg flutamide; FIN: 20 mg/kg finasteride.

In Figure 2, *Rxfp1* mRNA expression in the patellar tendon following 125 and 250 μg/kg testosterone treatment was lower than the control (*p <* 0.05). Administration of FLU to 125 and 250 μg/kg testosterone treated groups resulted in an increase in *Rxfp1* mRNA expression by approximately 0.69- and 0.86-fold respectively (*p <* 0.05). Finastride administration to these groups also resulted in 0.20- and 0.22-fold increase in *Rxfp1* mRNA expression respectively. The expression of *Rxfp2* mRNA in the patellar tendon in 125 and 250 μg/kg testosterone-treated groups following FLU administration was 0.30 and 0.71-fold higher respectively (*p <* 0.05). The expression of *Rxfp2* mRNA in the group receiving 125 and 250 μg/kg testosterone treatment plus FIN administration was also increased by 0.38- and 0.39-fold respectively (*p <* 0.05).

### 2.3. Rxfp1 and Rxfp2 Protein Expression in Patellar Tendon

**Figure 3.**
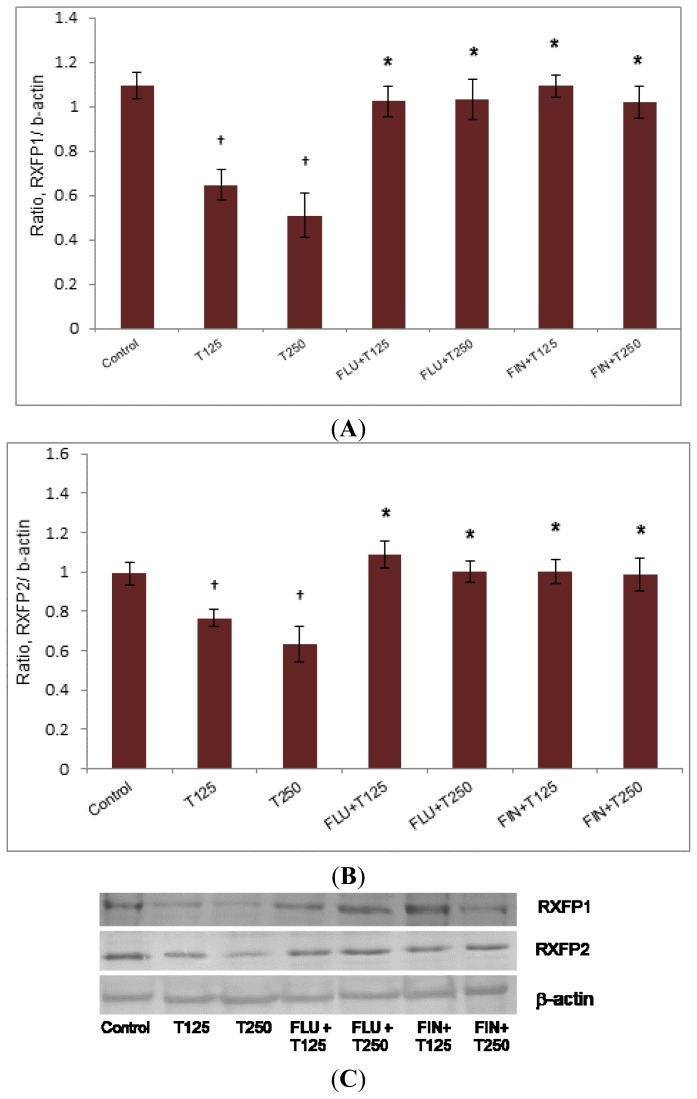
The expression of Rxfp1 (**A**) and Rxfp2 (**B**) protein in the patellar tissue homogenate from ovariectomised rats treated with testosterone with and without FLU and FIN. A decrease in Rxfp1 protein expression was noted following testosterone treatment which was antagonized by FLU and FIN. The expression of Rxfp2 protein was reduced following testosterone treatment which was also antagonized by FLU and FIN. The antagonizing effect of FIN indicated that DHT and not testosterone mediates the inhibition of Rxfp1 and Rxfp2 protein expression (**C**) (Data were expressed as mean ± SEM, *n =* 6 per treatment group; T125: 125 μg testosterone; T250: 250 μg testosterone; FLU: 10 mg/kg flutamide; FIN: 20 mg/kg finasteride). ^†^ *p <* 0.05 as compared to control; * *p <* 0.05 as compared to testosterone only treatment for the respective group.

In Figure 3, the expression of Rxfp1 protein in the patellar tendon was higher in the group receiving FLU with testosterone (0.79- and 0.83-fold increase respectively as compared to 125 and 250 μg/kg testosterone only treatment (*p <* 0.05). The expression of Rxfp1 protein in the groups receiving FIN with testosterone was also significantly increased as compared to without FIN (0.59- and 0.52-fold increase respectively as compared to 125 and 250 μg/kg testosterone only treatment) (*p <* 0.05). The expression of Rxfp2 protein in the patellar tendon was increased following treatment with FLU plus the two doses of testosterone, (0.29- and 0.36-fold increase respectively as compared to 125 and 250 μg/kg testosterone treatment alone) (*p <* 0.05). The expression of Rxfp2 protein was significantly increased following treatment with FIN plus testosterone as compared to testosterone only treatment (0.2- and 0.24-fold increase respectively as compared to 125 and 250 μg/kg testosterone) (*p <* 0.05).

### 2.4. Rxfp1 and Rxfp2 mRNA Expression in Lateral Collateral Ligament

**Figure 4.**
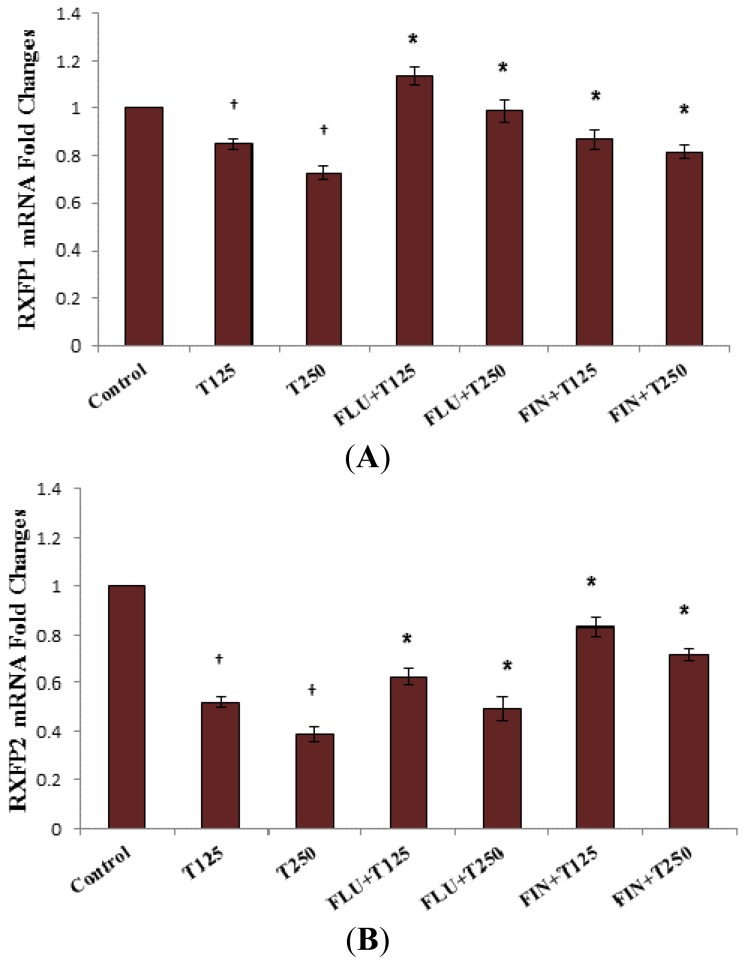
Changes in *Rxfp1* (**A**) and *Rxfp2* (**B**) mRNA expression in the lateral collateral ligament of steroid-replaced ovariectomised rats in the presence of FLU and FIN. Treatment with 125 and 250 μg/kg/day testosterone caused a decrease in *Rxfp1* and *Rxfp2* mRNA expression as compared to control (testosterone only treatment) (*p <* 0.05). Administration of flutamide and finasteride caused a significant increase in the expression of *Rxfp1* and *Rxfp2* with FLU inhibition was relatively greater than FIN for *Rxfp1*, however FIN inhibition was relatively higher than FLU for *Rxfp2* mRNA expression. ^†^ *p <* 0.05 as compared to control; * *p <* 0.05 as compared to testosterone only treatment for the respective group.

In Figure 4, the expression of *Rxfp1* mRNA in the lateral collateral ligament was reduced following treatment with testosterone at 125 and 250 μg/kg/day. Administration of FLU and FIN resulted in a significant increase in mRNA expression where effect of the latter suggested DHT-mediated testosterone effect. Meanwhile, decreased *Rxfp2* mRNA expression was also noted following testosterone treatment alone (0.31- and 0.33-fold decrease respectively following exposure to 125 and 250 μg/kg testosterone) which was also antagonized by FLU and FIN (*p <* 0.05).

### 2.5. Rxfp1 and Rxfp2 Protein Expression in Lateral Collateral Ligament

**Figure 5.**
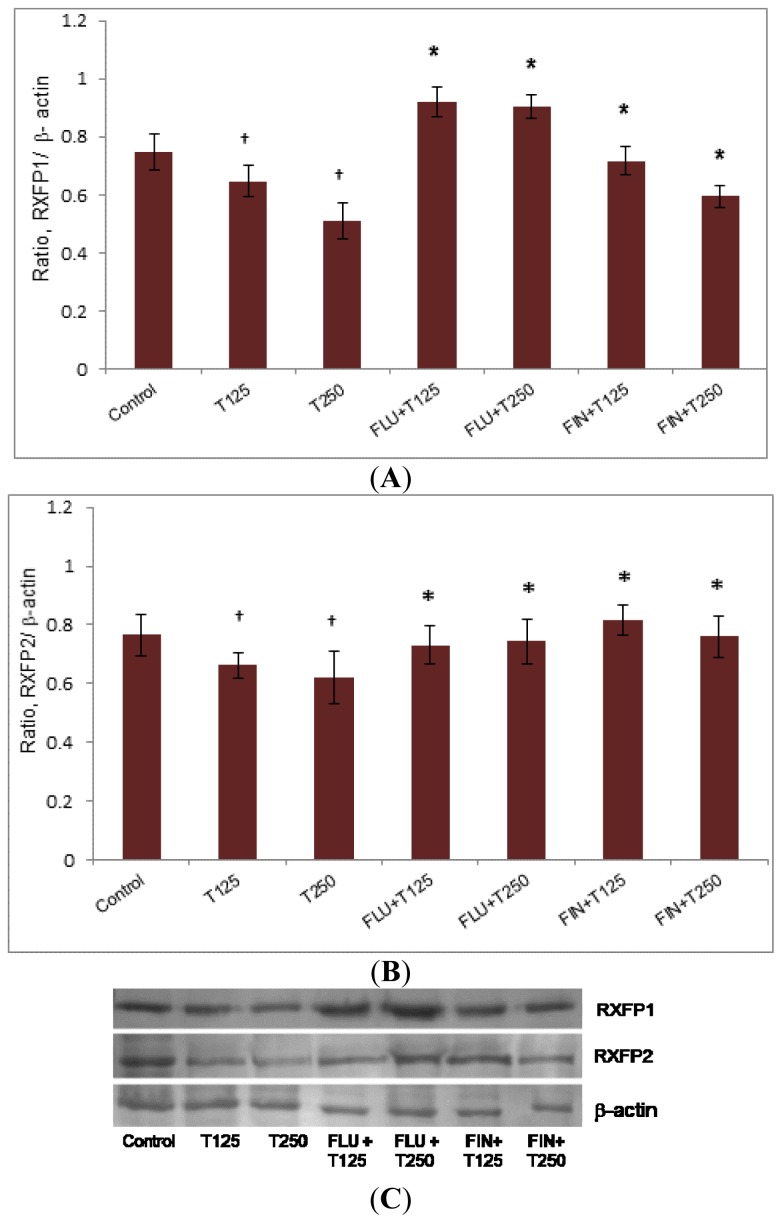
The expression of Rxfp1 (**A**) and Rxfp2 (**B**) protein from the homogenate of lateral collateral ligaments of ovariectomised rats treated with 125 and 250 μg/kg testosterone with and without FLU and FIN administration. A dose-dependent decrease in Rxfp1 protein expression was noted following treatment with both doses of testosterone which was antagonized by FLU and FIN. Similarly, a decrease in Rxfp2 protein expression was observed following treatment with both doses of testosterone which was antagonized by FLU and FIN (**C**). Data were expressed as mean ± SEM, *n =* 6 per treatment group; T125: 125 μg testosterone; T250: 250 μg testosterone; FLU: 10 mg/Kg FLU; FIN: 20 mg/kg FIN. ^†^ *p <* 0.05 as compared to control; * *p* and * *p <* 0.05 as compared to testosterone only treatment for the respective group.

In Figure 5, the expression of Rxfp1 protein in the lateral collateral ligament was reduced following testosterone only treatment, which was antagonized by FLU and FIN. FLU administration resulted in 0.28- and 0.48-fold increase in Rxfp1 expression as compared to 125 and 250 μg/kg testosterone only treatment respectively (*p <* 0.05). The reduced Rxfp1 expression following 125 and 250 μg/kg testosterone administration was antagonized by FIN (*p >* 0.05). A decrease in the expression of Rxfp2 protein in the lateral collateral ligament following administration of both doses of testosterone was also antagonized by FLU and FIN.

## 3. Discussion

To the best of our knowledge, this study is the first to demonstrate the effect of testosterone on knee laxity in an ovariectomised rat model. Ovariectomy was performed to investigate sex-steroid effect on the ligament laxity, as removal of endogenous hormone and replacement with exogenous steroid would ensure that the effect observed was due to the action of individual steroid rather than the combined effect of multiple steroids produced by the ovaries. In the female of reproductive age, 25% of the circulating testosterone originates from the ovaries while the remaining is derived from conversion of androsteinedione into testosterone in the adrenal gland [27]. In humans, the level of endogenous testosterone has been reported to fluctuate throughout the menstrual cycle which was the highest at around the time of ovulation [28]. A recent finding by O’Leary *et al*. [29] reported that a prolonged aerobic training in the eumenorrheic women induces a short-term elevation in the plasma testosterone levels, which appears to be unrelated to the level of estrogen and the phases of menstrual cycle. There is currently no report on changes in the testosterone level throughout the oestrous cycle.

Testosterone has been proposed to influence knee laxity in female. A positive correlation between plasma testosterone, free androgen index (FAI) and anterior cruciate ligament stiffness has been reported in young females near ovulation [25]. The mechanisms underlying testosterone effect on ligament laxity is unknown, however a limited finding in the prostate gland indicated that testosterone as opposed to estrogen affects collagen metabolism [30] via downregulating the expression of estrogen receptor (ER)-α [31]. So far, no study has reported testosterone effect on relaxin receptor expression in the joints, although relaxin has been shown to affect ligament laxity and its receptor was expressed in female knee joint of both humans [32] and rodents [7]. Our findings which revealed downregulation of relaxin receptor expression in the knee ligaments and tendons by testosterone has provided explanation for the observed decrease in knee passive ROM under testosterone influence. A decrease in knee passive ROM could be due to decreases in the relaxin effect on ligament, rendering the tissue to have decreased laxity.

Our study has provided the first direct evidence on testosterone effect on the passive ROM in rats’ knee. We have shown that testosterone reduces the ROM, which was inhibited by FLU and FIN. In the group receiving 125 and 250 μg/kg/day testosterone treatment, the presence of relaxin did not significantly increase knee passive ROM. This finding suggested that knee was not responsive to relaxin in the presence of testosterone due to relaxin receptor downregulation. The knee ROM was however increased following FLU or FIN administration, suggesting that inhibition of androgen binding to its receptor and conversion of testosterone to DHT caused the increase in knee laxity. FLU inhibition confirmed the release of DHT-mediated inhibition on relaxin receptor expression through androgen receptor binding. Relaxin administration resulted in further increase in knee laxity in the groups receiving both testosterone doses with FLU or FIN, which again suggested the release of DHT-mediated inhibition on relaxin receptor expression. Meanwhile, increased knee laxity in the groups receiving testosterone with FLU or FIN without relaxin suggested that endogenously produced relaxin from sources such as breast [33] might influence knee laxity.

The presence of AR has been reported in human female knee joint [25,34]. In this study, inhibition of testosterone-mediated down-regulation of Rxfp1 and Rxfp2 protein and mRNA expression in the patellar tendon and lateral collateral ligament by FLU confirmed AR involvement in mediating testosterone effect. Our findings have also confirmed DHT involvement in mediating testosterone action as evidenced from the antagonizing effect of FIN on testosterone-mediated decrease in knee passive ROM as well as testosterone-mediated down-regulation of Rxfp1 and Rxfp2 protein and mRNA expression in these knee structures. FIN is a competitive, selective, and reversible inhibitor of 5α-reductase, an enzyme that converts testosterone to DHT [35]. In females, DHT exerts multiple physiological effects mainly in the uterus and ovaries [36]. DHT has also been reported to cause an increase in the twitch and titanic contraction of fast-twitch skeletal muscle in mice [37]. Although we have shown evidence that DHT is most likely involved in causing a decrease in knee laxity, further studies are needed to support DHT participation such as identifying the expression and measuring the activity of 5α-reductase enzyme in the knee. In our study, a direct effect of DHT on knee laxity and relaxin receptor isoforms expression was unable to be investigated since this prohibited compound was not commercially available.

Our findings have important implications in the field of exercise physiology. Testosterone could affect female knee laxity as its level was reported to be the highest in the ovulatory phase of the menstrual cycle [25]. Although we have found that testosterone decreases knee laxity, the findings by Shultz *et al*. [23] who reported a positive correlation between plasma estrogen and testosterone level with knee laxity throughout the menstrual cycle suggested dominant effects of estrogen over testosterone. In female athletes, anabolic steroids are widely used to boost their physical performances. Despite reported adverse effects, this drug could help to strengthen the knee via its effect on ligament and tendon laxity, which could improve athletes’ short-term performance as well as reduce the risk of non-contact knee injury. Additionally, the strength of the muscles controlling knee joint movement was also increased [38]. This study has shown for the first time the direct effect of testosterone on knee passive ROM and Rxfp1 and Rxfp2 protein and mRNA expression in a rat model, which was mediated via DHT and involved androgen receptor binding. These therefore could explain the protective role of testosterone against non-contact knee injury in female. Precaution however is needed when extrapolating these data to humans since relaxin action on human joints might not be as pronounced as those observed in rodents [39].

## 4. Experimental Sections

### 4.1. Animal Preparation and Hormone Treatment

**Figure 6.**
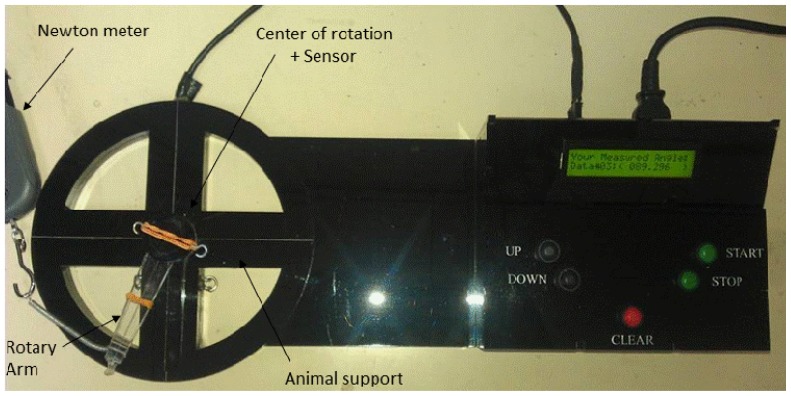
Measurement of rats’ knee ROM by a digital miniature goniometer (Patent IC/No: PI 2013701411) as shown above. The device consists of an arm that is attached to a sensor that detects changes in angle once the arm is rotated. The sensor is connected to the software that analyzes the angle using a Torque principle. Torque is defined as the tendency of a force to rotate an object around a fixed axis which is given by this formula: “τ = *r F*sinθ” formula. *r*: rat leg length (*r*); *F*: force (*A*); θ: angle between the applied force and rat’s ROM (A and B distance).

All procedures involving animals were approved by the Faculty of Medicine Animal Care and Use Committee (ACUC), University Malaya (UM), Kuala Lumpur, Malaysia with ethics number: FIS/22/11/2011/FD(R). Adult female Wistar-Kyoto (WKY) rats aged 8–10 weeks, weighing 180–200 g were obtained from the Animal House, Faculty of Medicine, University Malaya (UM), Kuala Lumpur, Malaysia and were caged in groups of six, in a clean and well ventilated standard environment of 12 h light: dark cycle. The animals had free access to a soy-free diet (Harlan diet, Blackthorn, UK) and tap water *ad libitum*. In the first part of the study, the rats were ovariectomized 14 days prior to steroid treatment to eliminate the effect of endogenous sex-steroids. Based on the treatment protocol, the animals were divided into different groups (*n =* 6 per group) (i) control group which received peanut oil injection only (vehicle); (ii) and (iii) testosterone at 125 μg/kg (T125) and 250 μg/kg (T250) [40]; (iv) T125 plus FLU; (v) T250 plus FLU; (vi) T125 plus FIN and (vii) T250 plus FIN. All drugs were subcutaneously injected behind the neck for three consecutive days. To determine the involvement of AR and 5-α reductase enzyme, androgen receptor blocker, FLU (Sigma Aldrich, St. Louis, MO, USA, at 10 mg/Kg) [41] and 5α reductase inhibitor, FIN (Sigma-Aldrich, St. Louis, MO, USA, at 20 mg/kg) [42] were subcutaneously injected 30 min prior to testosterone injection. Duplicate groups received subcutaneous injection of recombinant relaxin (Sigma-Aldrich, St Louis, MO, USA; cat number SRP3147-25UG) at 25 ng/kg also for three days. A day after the last day of treatment, rats were anaesthetized and knee passive ROM was determined using a digital miniature goniometer (Figure 6). At the end of measurement, animals were decapitated and patellar tendon and lateral collateral ligaments were harvested for Rxfp1 and Rxfp2 protein and mRNA expression.

### 4.2. Measurement of Knee ROM Following Sex-Steroid Treatments

Rat’s knee ROM was measured using a digital miniature goniometer as shown in Figure 6. A day after the last day of injection, rats were anesthetized using ketamine and xylazine (80 + 8 mg/kg). The depth of anesthesia was confirmed from lack of response to a painful stimuli, which was applied to the foot’s plantar surface [43]. It is important to maintain animals in deep anesthesia as to prevent active muscle contraction in response to painful stimuli, which will result in increased resistance towards the passive traction. Hip and knee joints were fixed and rested on the sensor. Meanwhile, the lower leg (knee up to ankle) was tied *in-situ* to the device arm. Knee passive ROM was measured by pulling the device arm in a clockwise direction at a minimum constant force of 12 ± 1 Newton (N) using a mini digital Newton meter (American Weight, Loiusville, KY, USA; model: AMW-SR-1KG). Once the force exceeded 13 N, traction was immediately terminated and the angle obtained was recorded which represents the passive knee extension. Determination of the angle was made in different groups of treatment.

### 4.3. *Rxfp1* and *Rxfp2* mRNA Expression Analyses by Real Time PCR (qPCR)

Patellar tendon and lateral collateral ligament were harvested from rat left hind limb and was kept in RNALater solution (Ambion, Calsbad, CA, USA) prior to RNA extraction. Total RNA was isolated from 30 mg tissues (wet weight) using RNeasy Mini Kit (Qiagen, Hilden, Germany). The RNA purity and concentration was assessed by 260/280 UV absorption ratios (Gene Quant 1300, Buckinghamshire, UK). The RNA was converted to cDNA using High Capacity RNA-to-cDNA Master Mix from Applied Biosystems, Foster City, CA, USA. One step Real Time PCR was performed to evaluate gene expression, with application of TaqMan^®^ RT-qPCR (Applied Biosystems, Foster City, CA, USA). *Hprt1* and *Gapdh* were used as reference genes. TaqMan primer and probe for *Rxfp1*, *Rxfp2*, *Gapdh* and *Hprt1* were obtained from predesigned assays from Applied Biosystems, Foster City, CA, USA. All experiments were carried out in three biological replicates. In Real Time PCR, the amplified region of cDNA was probed with a fluorescence-labeled probe. The assay used (TaqMan^®^-*Rxfp1*: Rn01495351; Lot No: 926762 and *Rxfp2*: Rn01412901; Lot No: 651878) amplifies a 116 bp segment of *Rxfp1* from the whole mRNA length of 2277 and 138 bp segment of *Rxfp2* from the whole mRNA length of 2214 bp. The catalogue number for the housekeeping genes are: *Gapdh*, Rn99999916_S1, Lot No: 10377343 which amplifies a 87 bp segment of *Gapdh* from the mRNA length of 1307 bp and *Hprt1*, Rn01527840, Lot No: 1118680 which amplifies a 67 bp segment from the mRNA length of 1260 bp. The target assay was validated *in-silico* using whole rat genome sequences and *in vitro* using whole rat cDNA sequences to ensure target sequences were detected (Applied Biosystems, Foster City, CA, USA). Therefore, the PCR product does not require additional sequencing for further validation. Real time-PCR program includes: 2 min at 50 °C reverse transcriptase, 20 s at 95 °C activation of polymerase, denaturation at 95 °C for 1 s and annealing at 60 °C for 20 s. Denaturation and annealing was performed for 40 cycles. Step one plus real time PCR machine, master mix and assays were purchased from Applied Biosystems, Foster City, CA, USA. Data was analyzed according to Comparative *C*_t_ (2^−ΔΔ^*^C^*^t^) method, in amplification of the target and of the reference genes were measured in the samples and reference. Measurements were normalized using GenEx software (bioMCC, Freising-Weihenstephan, Germany). The relative quantity of target in each sample was determined by comparing the normalized target quantity in each sample to the normalized target quantity in the reference. Data Assist v3 software from Applied Biosystems, Foster City, CA, USA was used to calculate the RNA folds changes.

### 4.4. Protein Expression Analysis by Western Blotting

Patellar tendon and lateral collateral ligament from rat’s left hind leg were separated from fat and rinsed with 0.1% phosphate buffer solution. The tissues were then snapped frozen in liquid nitrogen and stored at −80 °C prior to protein extraction. Total amount of protein was extracted from 50 mg (wet weight) tissue. After extraction of total protein with PRO-PREP (Intron, Seoul, Korea), equal amount of protein from each tissue lysate were mixed with a loading dye, boiled for five min and separated by SDS-PAGE 10%. The protein was then transferred onto a PVDF membrane (BIORAD, Hercules, CA, USA) and blocked with 5% BSA for 90 min at room temperature. The membrane was exposed for 90 min to rabbit polyclonal primary Rxfp-1/LGR7 antibody (Abcam, Cambridge, UK), mouse polyclonal Rxfp2/LGR8 (Abcam, Cambridge, UK) and rabbit anti-mouse beta actin (Abcam, Cambridge, UK) diluted at 1:1000 in PBS containing 1% BSA and Tween 20. Blots were washed three times each for five min, and incubated with anti-rabbit and anti-mouse horseradish peroxidase conjugated secondary antibody (Santa Cruz Biotechnology, Dallas, TX, USA) at a dilution of 1:2000, for 1 h. The membrane was then washed and subjected to Opti-4CN™ Substrate Kit from Bio-Rad (Hercules, CA, USA) to visualize the protein bands. Photos of the blots were captured using a gel documentation system and the density of each band was determined using Image J software. The ratio of each target band/β actin was calculated using Image J version 1.46j (National Institutes of Health, Bethesda, MD, USA) and was considered as the expression level of the target proteins.

### 4.5. Statistical Analysis

For knee range of motion, real time PCR, and Western blotting, six rats per treatment group were used. The density of protein bands in Western blotting was analyzed using Image-J software, and the results were presented as a ratio of the target protein to β-actin. Statistical differences were evaluated by analysis of variance (ANOVA) followed by Duncan’s new multiple-range test and Student’s *t-*test. A probability level of less than 0.05 (*p <* 0.05) was considered to be significantly different. *Post-hoc* statistical power analysis was performed for all the experiments conducted and all values obtained were >0.8 which were considered as adequate. Meanwhile, the Shapiro-Wilk test was performed to test for data normality and all values obtained were >0.05 which indicated that these data were normally distributed.

## 5. Conclusions

In conclusion, our study has demonstrated that testosterone-related decrease in knee laxity is mediated via its metabolite, 5α-dihydrotestosterone and is mediated via binding to the androgen receptor. This therefore provides molecular basis underlying testosterone effect on knee laxity.
